# Unveiling insights with ChatGPT: A dialogue on deep vein thrombosis

**DOI:** 10.21542/gcsp.2024.30

**Published:** 2024-08-01

**Authors:** Rutva Jani

**Affiliations:** C U Shah Medical College and Hospital, Dudhrej Road, Surendranagar, Gujarat, 363001, India

Dear Editor,

Developed by OpenAI, ChatGPT is engineered to be exceptionally smart and user-friendly, capable of grasping and replying to intricate queries in a way that feels natural and human-like. With its advanced capabilities, ChatGPT is transforming our engagement with technology and leading the charge toward a future of innovative, conversational AI^1^.

Large language models (LLMs) are specialized AI models designed to understand and generate text in a human-like manner. Their primary goal is to comprehend the structure, syntax, semantics, and contextual nuances of natural language, enabling them to produce coherent and contextually appropriate responses^2^.

One way that AI and language models can assist in cardiology education and training is by offering access to educational content and resources. They can review medical textbooks, research articles, and other resources to provide healthcare providers with up-to-date information on cardiovascular diseases and treatments. This allows clinicians to stay informed about recent developments in cardiology, ensuring they can offer the best possible care to their patients^3^.

Deep vein thrombosis (DVT) is characterized by the formation of blood clots in the deep veins, often occurring in the legs. DVT in the lower limbs can lead to considerable health complications and may develop into conditions such as pulmonary embolism and post-thrombotic syndrome^4^. With the rapid adoption of new technologies and AI tools like ChatGPT, individuals, particularly those who are tech-savvy, might seek fast and accessible information regarding DVT on digital forums.

**Table 1:** 

**Question**	**Response by ChatGPT**
What exactly is DVT?	Deep Vein Thrombosis, or DVT, is when a blood clot forms in a vein deep inside your body, usually in the lower leg, thigh, or pelvis. It’s like a traffic jam in your veins where blood cells stick together and block the flow.
What causes DVT?	• Prolonged Immobility: Sitting still for extended periods, such as during long flights or bed rest, can impede blood flow. Reduced movement allows clots to form. • Surgical Procedures: Surgeries can damage blood vessels, triggering clot formation. Postoperative immobility further exacerbates the risk. • Medications: Certain drugs can thicken the blood, increasing the likelihood of clot development. Examples include hormonal contraceptives and hormone replacement therapy. • Smoking: Tobacco use damages blood vessels and promotes clotting. Smokers face an elevated DVT risk. • Obesity: Excess weight strains veins, hindering blood flow. Obesity is a significant risk factor. • Genetics: Sometimes, DVT runs in families. A genetic predisposition may contribute to clot formation.
What are the symptoms? How would I know I have it?	Swelling and Pain: DVT often causes localized swelling in the affected leg. The leg may feel heavy, tight, or uncomfortable. Pain can range from mild discomfort to intense throbbing. Tenderness: When you touch the affected area, it might be tender or sore. The skin over the clot can feel sensitive. Redness and Warmth: DVT can cause the skin to appear redder than usual. The affected leg may feel warm to the touch due to inflammation. Silent DVT: Surprisingly, some individuals experience no noticeable symptoms. Silent DVT can be dangerous because it remains undetected until complications arise. Being vigilant about these symptoms and seeking medical evaluation promptly can prevent serious complications associated with DVT.
What happens when someone has DVT? How is it treated?	If someone has Deep Vein Thrombosis (DVT), the primary goal of treatment is to prevent the clot from getting bigger, breaking loose, and traveling to the lungs, where it can cause a pulmonary embolism. The most common treatment for DVT is anticoagulant medication, also known as blood thinners. These growing and new ones from forming. In some cases, doctors may use thrombolytic therapy, which involves injecting clot-dissolving medication directly into the clot. Another treatment option is the placement of a vena cava filter, a small device inserted into the inferior vena cava (a large vein that carries blood from the lower body to the heart) to catch any clots that break loose and prevent them from reaching the lungs.
How long does treatment for DVT last?	The duration of treatment for Deep Vein Thrombosis (DVT) is not fixed and can vary depending on the individual case. For instance, if it is a first-time occurrence of DVT and there are no complicating factors, the treatment typically lasts for three to six months. However, if the DVT is caused by a temporary risk factor, such as surgery, the treatment duration maybe shorter. On the other hand, for individuals with recurrent DVT or ongoing risk factors such as cancer, the treatment may be longer or even indefinite. It is important to note that the exact duration of treatment should be personalized based on the individual’s risk factors and response to therapy.
Who is at risk for DVT?	There are several factors that can increase an individual’s risk of developing Deep Vein Thrombosis (DVT). These include: •** Age:** People over the age of 60 are at higher risk, though DVT can occur at any age. •** Family History:** Those with family history of clotting disorders are at an increased risk. •** Previous DVT:** Individuals who have had DVT before are at a higher risk of developing it again. •** Pregnancy and childbirth:** Pregnant women and women who have recently given birth are at an increased risk. •** Cancer:** People with certain cancers or undergoing cancer treatments are at a higher risk. •**Heart disease or stroke:** Those with a history of history of heart disease or stroke are at an increased risk. •** Obesity:** People who are overweight or obese are at a higher risk. It is important to note that having one or more of these risk factors does not necessarily mean that an individual will develop DVT, but it does increase their likelihood.
How can we prevent it?	There are several measures that can be taken to prevent Deep Vein Thrombosis (DVT). One of the most important things is to avoid sitting still for long periods of time, especially during long trips. It is recommended to get up and move around every now and then to keep the blood flowing. Staying hydrated and exercising regularly can also help prevent DVT. For individuals who are at risk, doctors might suggest wearing special compression socks that squeeze the legs to keep the blood moving. In some cases, medication to prevent clots may also be prescribed. It is important to consult with a doctor to determine the best prevention measures for each individual case.
What lifestyle changes can reduce the risk of DVT?	Making certain lifestyle changes can significantly reduce the risk of developing Deep Vein Thrombosis (DVT). These include: •** Exercising regularly:** Regular physical activity helps improve blood flow and reduce the chances of clot formation. •** Losing weight:** If you’re overweight, losing weight can help reduce the pressure on the veins in your legs and pelvis, decreasing the risk of DVT. •** Quitting smoking:** Smoking affects blood clotting and circulation, increasing your risk of DVT. Quitting smoking can help reduce this risk. •** Staying hydrated:** Staying hydrated, especially during long trips, can help prevent blood from thickening, reducing the risk of clot formation. •** Avoiding long periods of immobility:** If you’re traveling or have a sedentary job, it’s important to take breaks to stand up, stretch, and walk around to keep the blood flowing and reduce the risk of DVT. • **Following a healthy diet:** Eating a healthy diet that’s low in saturated fat and rich in fruits, vegetables, and whole grains can help improve overall vascular health and reduce the risk DVT. It is important to consult with a doctor to determine the best prevention measures for each individual case.
What are the complications?	Deep Vein Thrombosis (DVT) can have serious complications if left untreated. One of the most serious complications is pulmonary embolism, which occurs when a clot breaks free and travels to the lungs, causing a blockage. This can be life-threatening and requires immediate medical attention. Another potential complication of DVT is the development of long-term problems in the affected leg, such as pain, swelling and even ulcers. These complications can significantly impact an individual’s quality of life and may require ongoing management. It is important to seek prompt treatment for DVT to reduce the risk of these complications.
Can DVT be completely cured?	Deep Vein Thrombosis (DVT) is typically treatable with a good prognosis when caught early and managed well. Most people recover from DVT with appropriate treatment, which involves medication to prevent the clot from growing and to reduce the risk of further clotting. However, some may develop post-thrombotic syndrome, which can cause long-term symptoms like swelling, pain, and ulcers in the affected leg. Although the initial clot can be resolved, it’s important to address the underlying risk factors to prevent new DVTs from occurring. This means that while the condition can be effectively managed, it may not be completely cured in all cases, and ongoing management may be necessary to prevent reoccurrence. It is important to note that the treatment and management of DVT is highly individualized and depends on factors such as the size and location of the clot, the presence of any underlying medical conditions, and the individual’s overall health. In some cases, the clot may dissolve on its own with the help of blood thinners, while in others, more aggressive treatment such as thrombolytic therapy or the placement of a vena cava filter may be necessary. Ongoing management may involve lifestyle changes such as losing weight, quitting smoking, and staying active to reduce the risk of reoccurrence. It is important to work closely with a healthcare provider to develop an individualized treatment plan.
Is DVT life-threatening?	If left untreated, DVT can have serious complications. One of the most serious complications is pulmonary embolism, which occurs when a clot breaks free and travels to the lungs, causing a blockage. This can be life-threatening and requires immediate medical attention. A pulmonary embolism is a medical emergency and can be fatal. That’s why it’s so important to get treated if you think you might have DVT. Prompt treatment can help prevent the clot from breaking loose and traveling to the lungs, reducing the risk of a pulmonary embolism.

Table 1 describes a conversation comprising of eleven questions, between a common man and ChatGPT about DVT.

Through our assessment, it was found that ChatGPT provided extensive and accurate descriptions in a straightforward manner. For example, it explained how prolonged immobility could increase the risk of developing DVT and emphasized the unpredictability of completely avoiding DVT but suggested preventative measures like regular exercise and hydration. ChatGPT also advised consulting healthcare professionals for accurate diagnosis and treatment plans, including common interventions for DVT management.

ChatGPT’s interactions with individuals seeking advice on DVT show a level of empathy in its responses. However, the lack of verification for these responses presents a challenge, as there are no direct citations of sources or references to back the provided information. This issue highlights a significant limitation in ensuring the accuracy and authenticity of the guidance offered.

Language models like ChatGPT show immense potential in clinical information dissemination but are limited by their non-deterministic response generation to similar queries. Despite these limitations, ChatGPT could aid vascular specialists in promptly delivering empathetic, high-quality information *via* online platforms. Specialists can enhance the accuracy and reliability of this information by cross-referencing with authoritative medical literature.

Future research efforts should evaluate the dependability and precision of the responses ChatGPT offers to queries related to DVT. An alliance of vascular specialists with an interest in artificial intelligence could create an innovative AI tool based on LLMs. This tool would produce verified, high-quality, and reliable responses to patient inquiries, marking a significant advancement in patient education and online consultations.
